# The United States College and the Three-years’ Course

**Published:** 1895-05

**Authors:** 


We learn with much regret that the United States College of Veterinary Surgeons will announce a change from a three-years to a two-years' course, commencing with the session of 1895–96. This is a step backward; and we are sure will prove a serious blow to this school, which stepped out on so firm and determined a basis.

